# Genome sequence of *Lacticaseibacillus paracasei* G277-1.1, a clinical strain that forms enhanced biofilms with *Streptococcus mutans*

**DOI:** 10.1128/mra.00481-25

**Published:** 2025-08-25

**Authors:** Xiaochang Huang, Hemendra Pal Singh Dhaked, Yihong Li, Zezhang T. Wen

**Affiliations:** 1 State Key Laboratory of Food Science and Technology, Nanchang University 47861, Nanchang, Jiangxi, China; 2 Department of Oral and Craniofacial Biology, School of Dentistry, Louisiana State University Health Sciences Center 12258, New Orleans, Louisiana, USA; 3 Department of Public and Ecosystem Health, Cornell University 5922, Ithaca, New York, USA; 4 Department of Microbiology, Immunology and Parasitology, School of Medicine, Louisiana State University Health Sciences Center 12258, New Orleans, Louisiana, USA; Wellesley College, Wellesley, Massachusetts, USA

**Keywords:** *Lacticaseibacillus paracasei*, genome sequence, dental caries, *S. mutans*

## Abstract

*Lacticaseibacillus paracasei* G277-1.1 was isolated from dental plaque of a patient with severe early childhood caries. It forms synergistic biofilms with *Streptococcus mutans in vitro*. Here, we report its high-quality genome sequence, revealing insights on its inter-species interaction with *S. mutans*.

## ANNOUNCEMENT

*Lacticaseibacillus paracasei* G277-1.1 was isolated from a plaque sample, which was collected from a carious lesion of a patient with severe childhood caries using a regular dental bur and grown in lactobacillus selection LBS medium (Dickinson and Company, Sparks, MD) in New York, NY (1). It forms limited biofilms when growing alone *in vitro*, but its biofilm formation can be significantly enhanced when co-cultured with *Streptococcus mutans* in biofilm medium containing 18 mM glucose and 2 mM sucrose in an aerobic chamber with 5% CO_2_. Here, we present the complete genome sequence of G277-1.1, which will help us study the pathophysiology of this cariogenic bacterium and the molecular mechanism of its inter-species interaction with *S. mutans* under the conditions studied.

For genomic DNA extraction, strain G277-1.1 was grown in MRS broth at 37°C until mid-exponential phase, and its genomic DNA was extracted using the ZymoBIOMICS DNA Miniprep Kit. For sequencing, 3.5 µg of genomic DNA was sheared using the Megaruptor three system, and the sheared DNA was processed into a barcoded SMRTbell library using the PacBio SMRTbell prep kit 3.0 (PacBio.com, PN: 102-182-700) as recommended. Post-library construction, binding, cleanup, and polymerase treatment were performed with the Revio reagent kit (PacBio.com, PN: 102-739-100) as instructed by the manufacturer, and followed by sequencing on a PacBio Revio platform. Raw sequencing data underwent initial processing with Lima (https://github.com/PacificBiosciences/pbbioconda) for deduplication, adapter trimming, and quality control, after which BAM files were converted to FastQ format via SamTools (2). For genome assembly, PacBio HiFi reads were analyzed using Flye (v2.9.2) with parameters set as --ASM-coverage 50—genome size 6 Mbp -pacbio hifi read-error 0.02 (3). Contigs were circularized using Circlator (v1.5.5) to achieve a complete chromosome (4). The finalized assembly was functionally annotated with the NCBI Prokaryotic Genome Annotation Pipeline (v6.10) (5) under default settings, and assembly quality metrics (6), including contiguity and completeness, were assessed using QUAST (v5.2.0) (7). Sequencing reads and genome assembly statistics are summarized in Table 1.

**TABLE 1:** Statistics of sequencing reads and genome assembly

Reads statistics				Assembly statistics			
Num_seqs	Sum len	Avg len	N50	Contigs_num	Largest contig	Total len	GC (%)
1,413,332	12,344,554,469	8,734	10,260	1	3,110,432	3,110,432	46.34

The genome of G277-1.1 is 3,114,032  bp long with a GC content of 46.34% (Table 1). It comprises 2,851 coding genes and 77 RNA genes, including three ncRNAs. Further analysis using antiSMASH V5.1.2 with default parameters (8) also revealed the core biosynthetic cluster genes included those encoding Class II bacteriocins, along with a gene cluster corresponding to the ATP-binding component of an ABC superfamily peptide transporter. These genes are often associated with interspecies interactions (9, 10), although it awaits further investigation if any of them are involved in G277-1.1’s synergistic biofilm formation with *S. mutans*.

## Data Availability

The assembly genome has been deposited in GenBank under the BioProject ID PRJNA665774, the GenBank accession number for the genome is CP186551. The BioSample number is SAMN47742411. The PacBio read accession number is SRR33296893. https://dataview.ncbi.nlm.nih.gov/object/57045657.
